# Royal Society of London

**Published:** 1861-07

**Authors:** 


ROYAL SOCIETY OF LONDON.—Dr. Brown-Séquard recently delivered the Croonian lecture. The subject was: "On the Relations between Muscular Irritability, Cadaveric Rigidity, and Putrefaction."

